# Metaorganismal choline metabolism shapes olfactory perception

**DOI:** 10.1016/j.jbc.2023.105299

**Published:** 2023-09-28

**Authors:** William J. Massey, Kristen E. Kay, Thomas C. Jaramillo, Anthony J. Horak, Shijie Cao, Lucas J. Osborn, Rakhee Banerjee, Marko Mrdjen, Michael K. Hamoudi, Daniel J. Silver, Amy C. Burrows, Amanda L. Brown, Ofer Reizes, Justin D. Lathia, Zeneng Wang, Stanley L. Hazen, J. Mark Brown

**Affiliations:** 1 Department of Cardiovascular and Metabolic Sciences, Lerner Research Institute, Cleveland Clinic, Cleveland, Ohio, USA; 2 Center for Microbiome & Human Health, Cleveland Clinic, Cleveland, Ohio, USA; 3 Rodent Behavior Core, Lerner Research Institute, Cleveland Clinic, Cleveland, Ohio, USA; 4 Department of Cancer Biology, Lerner Research Institute, Cleveland Clinic, Cleveland, Ohio, USA; 5 Cleveland Clinic Lerner College of Medicine of Case Western Reserve University, Cleveland, Ohio, USA; 6 Case Comprehensive Cancer Center, Case Western Reserve University, Cleveland, Ohio, USA; 7 Department of Cardiovascular Medicine, Heart Vascular and Thoracic Institute, Cleveland Clinic, Cleveland, Ohio, USA

**Keywords:** Nutrition, gut microbiome, metabolism, olfaction

## Abstract

Microbes living in the intestine can regulate key signaling processes in the central nervous system that directly impact brain health. This gut–brain signaling axis is partially mediated by microbe-host–dependent immune regulation, gut-innervating neuronal communication, and endocrine-like small molecule metabolites that originate from bacteria to ultimately cross the blood–brain barrier. Given the mounting evidence of gut-brain crosstalk, a new therapeutic approach of “psychobiotics” has emerged, whereby strategies designed to primarily modify the gut microbiome have been shown to improve mental health or slow neurodegenerative diseases. Diet is one of the most powerful determinants of gut microbiome community structure, and dietary habits are associated with brain health and disease. Recently, the metaorganismal (i.e., diet-microbe-host) trimethylamine N-oxide (TMAO) pathway has been linked to the development of several brain diseases including Alzheimer’s, Parkinson’s, and ischemic stroke. However, it is poorly understood how metaorganismal TMAO production influences brain function under normal physiological conditions. To address this, here we have reduced TMAO levels by inhibiting gut microbe-driven choline conversion to trimethylamine (TMA), and then performed comprehensive behavioral phenotyping in mice. Unexpectedly, we find that TMAO is particularly enriched in the murine olfactory bulb, and when TMAO production is blunted at the level of bacterial choline TMA lyase (*CutC/D*), olfactory perception is altered. Taken together, our studies demonstrate a previously underappreciated role for the TMAO pathway in olfactory-related behaviors.

The development of the mammalian brain initiates in early embryogenesis and extends through early adulthood. It is an elaborate and tightly regulated process involving both intrinsic and extrinsic cues. Locally derived cues originating within the central nervous system, in concert with signals originating outside of the brain, orchestrate long-distance migration of neural progenitor cells to specific regions or layers of the brain (1, 2, 3). Along with spatial migration across vast expanses within the brain, neural cells also extend their cell processes across long distances (sometimes hundreds of cell body diameters) to create signaling circuits that contribute to behavior (3, 4). Given that these pathways are constantly adjusted and refined throughout the lifetime of an organism, there is ample opportunity for environmental factors to influence neural circuitry. Recent evidence suggests that signals originating from the intestine can influence many aspects of brain health and disease by shaping the formation of the blood–brain barrier (BBB) (5, 6), neurogenesis (7, 8), myelination (9, 10, 11), microglia maturation/activation (12, 13, 14), and behavior (15, 16, 17, 18, 19). This so-called “gut-brain axis” has been associated with almost every neurological disorder including anxiety (20, 21), depression (17, 22), cognitive decline (23, 24), autism spectrum disorder (25, 26, 27), Parkinson’s disease (28, 29, 30), and Alzheimer’s disease (31, 32) as well as the extent of ischemic stroke (33, 34, 35). Our gut is the most expansive portal for environmental factors (diet, bacteria, viruses, fungi, among others) to interact with host genetic determinants of disease. In particular, there is a large body of emerging evidence that our gut microbiome may influence both developmental processes and maladaptive disease-associated pathologies in the brain (36, 37, 38, 39). However, mechanisms by which gut-brain axis crosstalk can influence brain health and disease are poorly understood.

There are at least three basic mechanisms by which the gut microbiota can influence processes in the central nervous system (1): gut microbes can direct neuronal communication between the enteric nervous system and the brain (2), indirect alterations in the host immune system driven by gut microbes, or (3) gut-brain endocrinology whereby bacterial products or metabolites can cross the BBB (36, 37, 38, 39). Collectively, these synergistic modes of communication can effectively link our environmental exposures (diet, xenobiotics, infectious agents, etc.) to brain health or disease. Recent evidence suggests that a diet-microbe-host metabolic pathway can produce an age-related small molecule metabolite called trimethylamine N-oxide (TMAO) that is elevated in patients with Alzheimer’s disease (40, 41, 42, 43), Parkinson’s disease (44, 45), and ischemic stroke (46, 47, 48, 49, 50, 51). Production of TMAO occurs *via* a meta-organismal metabolic pathway (52, 53, 54, 55, 56, 57, 58, 59, 60) that is initiated when TMA-containing nutrients present in high-fat foods or red meat (*i.e.*, phosphatidylcholine, choline, and carnitine) are metabolized by the primary gut microbial choline utilizing enzyme complex *CutC/D* (59) to generate trimethylamine (TMA), which is then further metabolized by the host liver enzyme flavin-containing monooxygenase 3 (FMO3) (60) to produce TMAO. Although TMAO has been most actively studied in the context of thrombosis and cardiovascular disease (CVD) (52, 53, 54, 55, 56, 57, 58), several studies have detected TMAO in either cerebrospinal fluid (40, 43) or brain tissue (61) indicating that this gut microbe-associated metabolite can cross the BBB or be produced locally in the brain. Because TMAO production is initiated in the gut, yet the end product metabolite TMAO is abundant in the central nervous system, it remains possible that metaorganismal TMAO production could serve as a potential link between diet, the gut microbiome, and neurological dysfunction.

Although TMAO can be detected in cerebrospinal fluid (CSF) and brain tissue (40, 43, 61), and elevated plasma TMAO levels are associated with age-related brain disorders such as Alzheimer’s disease, Parkinson’s disease, as well as ischemic brain injury (40, 41, 42, 43, 44, 45, 46, 47, 48, 49, 50, 51), the potential role of TMAO in shaping normal physiological brain function is not well understood. To address this, here we examined the regional abundance of TMAO in the mouse brain and coupled this to deep behavioral phenotyping in young healthy mice where TMAO production was inhibited pharmacologically. Our results highlight the importance of TMAO in the olfactory bulb and olfactory-related behavior. These studies provide a framework for understanding how therapeutic targeting of the gut microbial TMAO pathway could selectively impact host behavioral phenotypes that are associated with neurodevelopmental and neurodegenerative disorders. This work also provides new insights into how diet–microbe–host interactions that span the gut–brain axis may impact olfactory perception and olfactory-related behaviors.

## Results

### TMAO pathway metabolites are differentially abundant in mouse brain regions

**Figure 1:**
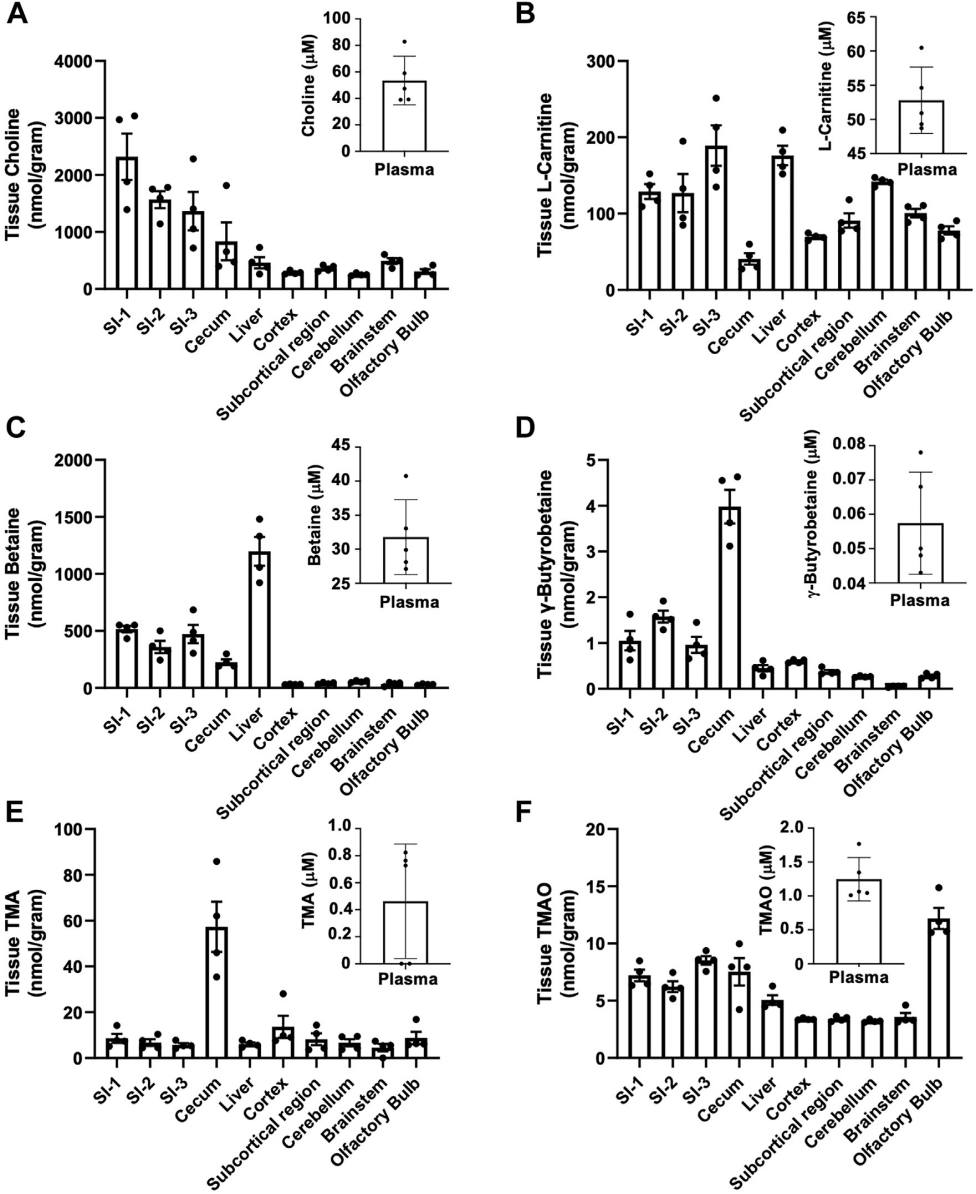
**Trimethylamine N-Oxide (TMAO) pathway metabolites are differentially abundant across the gut-liver-brain axis.** Tissue levels of TMAO pathway co-metabolites were quantified using stable isotope dilution liquid chromatography-tandem mass spectrometry (LC-MS/MS) in the gut, liver, and distinct brain regions. Tissues were harvested from male C57Bl/6J mice including intestinal segments including duodenum (SI-1), jejunum (SI-2), ileum (SI-3), cecum, liver, and different brain regions including cortex, subcortical region, cerebellum, brainstem, and olfactory bulb. Metabolites quantified included (*A*) choline, (*B*) L-carnitine, (*C*) betaine, (*D*) γ-butyrobetaine, (*E*) trimethylamine (TMA), and (*F*) trimethylamine N-oxide (TMAO). Data are presented as mean ± SEM from *n* = *4* mice.

**Figure 2:**
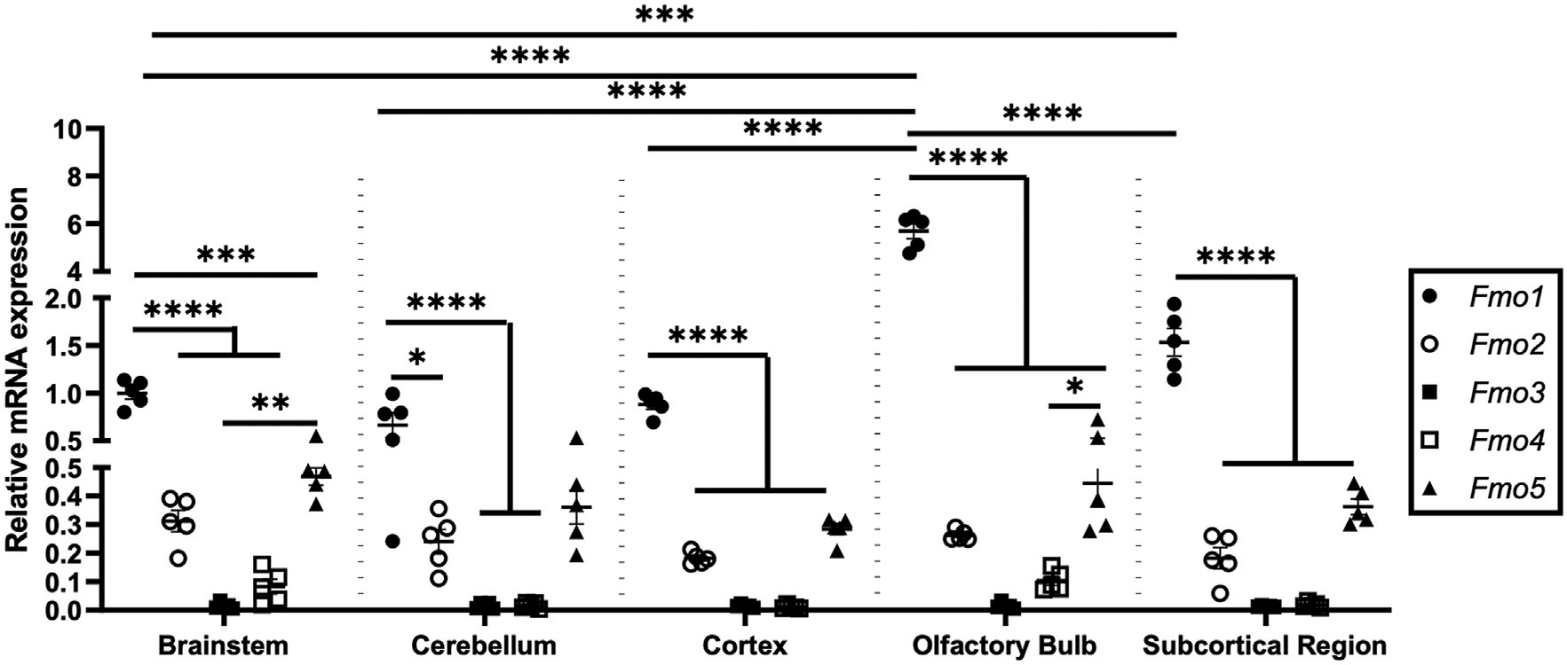
**Regional Differences in *Fmo1-5* expression in the mouse brain.** Brain regions (Brain stem, Cerebellum, Cortex, Olfactory Bulb, and Subcortical Region) were harvested from male C57Bl/6J, and expression of *Fmo1-5* transcripts was quantified using qRT-PCR. Data are presented as mean + SEM from *n* = 5 mice; ∗*p* < 0.05; ∗∗*p* < 0.01; ∗∗∗*p* < 0.001; ∗∗∗∗*p* < 0.0001.

While it has been demonstrated that TMAO can be readily detected in mouse brain throughout development (61), the regional distribution of TMAO pathway metabolites in mouse brain has never been established. To address this, we rapidly collected and extracted brain regions and quantified the tissue level of TMAO pathway metabolites as they related to levels in the intestine and liver where the TMAO pathway has been heavily studied (Fig. 1). It is important to note that all TMAO pathway co-metabolites examined (TMA, TMAO, choline, *L*-carnitine, betaine, and γ-butyrobetaine) were readily detectable in different brain regions as well as the gut and liver (Fig. 1, *A*–*F*). However, we found that out of all brain regions examined, TMAO was particularly enriched in the olfactory bulb where levels were nearly double that found in the liver (Fig. 1*F*). All other TMAO pathway metabolites (TMA, choline, *L*-carnitine, betaine, and *γ*-butyrobetaine) showed similar levels across different brain regions, with a slight enrichment of *L*-carnitine in the cerebellum (Fig. 1, *A*–*E*). Although it has long been assumed that TMAO production is most active in the liver (60), these data suggest that the murine olfactory bulb may be an additional site of TMAO production and/or accumulation. Aligned with this concept, quantitation of murine *Fmo1-5* transcripts across brain regions indicates that *Fmo1* is the most highly expressed of these transcripts in all brain regions analyzed but especially in the olfactory bulb (Fig. 2). Taken together these data suggest that, if TMAO is produced locally in the brain, it likely occurs independent of FMO3.

### Mice with low gut microbial TMA production have altered olfactory perception

**Figure 3:**
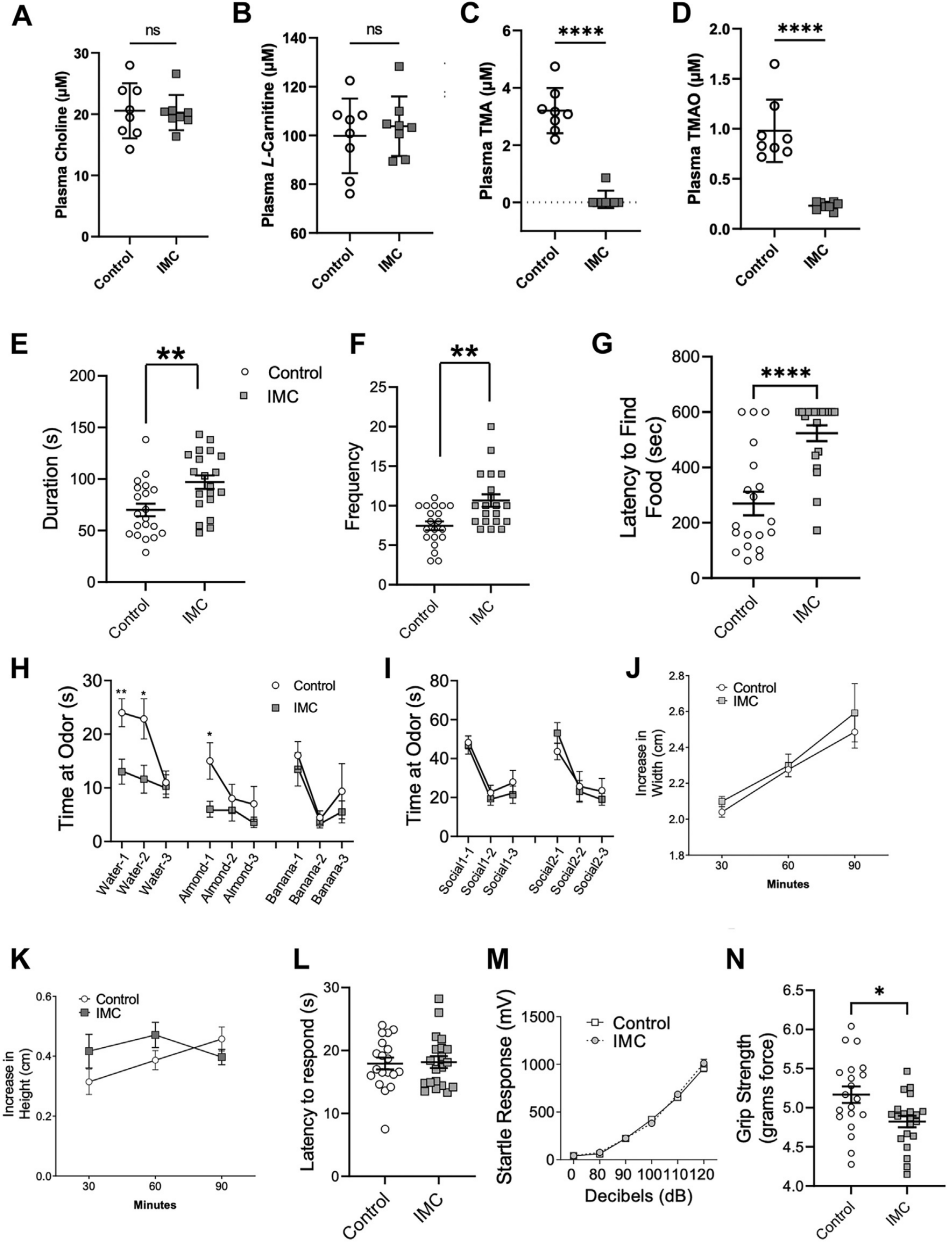
**Gut microbe-targeted choline trimethylamine lyase inhibition alters innate behaviors including olfactory perception.** Wild-type female C57BL/6J mice were treated with or without the gut microbial CutC/D inhibitor iodomethylcholine (IMC) in the drinking water and then subjected to a series of behavioral phenotyping tests to broadly assess alterations in innate behaviors. *A*–*D*, Plasma levels of trimethylamine N-oxide (TMAO) pathway co-metabolites (*A*) choline, (*B*) L-carnitine, (*C*) trimethylamine (TMA), and (*D*) TMAO were quantified *via* LC-MS/MS. Innate behavioral phenotyping tests included (*E* and *F*) grooming test, (*G*) olfactory cookie test, (*H*) food odor discrimination tests, (*I*) novel mouse odor discrimination test, (*J* and *K*) nest building test, (*L*) hotplate sensitivity test, (*M*) startle test, and (*N*) forepaw grip strength test. Data are presented as mean ± SEM; ∗*p* < 0.05; ∗∗*p* < 0.01; ∗∗∗*p* < 0.001; ∗∗∗∗*p* < 0.0001; *n* = 8/group for metabolomics, *n* = 20 control, *n* = 20 IMC-treated for grooming test, all other tests had *n* = 19 control, *n* = 20 IMC-treated.

In parallel to understanding the regional differences in TMAO pathway metabolites in mouse brain, we also wanted to understand the potential for the TMAO pathway to alter behavioral phenotypes that associate with these same brain regions. To understand the potential for the TMAO pathway to broadly impact brain function, we treated mice with a gut microbe-targeted small molecule inhibitor of CutC/D, iodomethylcholine (IMC) to lower circulating TMAO levels, and then performed a large battery of behavioral phenotyping tests. As we have previously demonstrated (58), IMC treatment effectively lowered plasma TMA and TMAO levels (TMA – *p* < 0.0001, TMAO – *p* < 0.0001; Fig. 3, *C* and *D*), without significantly altering key substrates for TMA production such as choline or *L*-carnitine (Fig. 3, *A*–*D*). In the first set of behavioral tests, we examined the effects of pharmacologic inhibition of TMAO production on several well-defined innate behaviors (Fig. 3, *E*–*N*). Innate behaviors in mice are genetically hardwired, do not require prior experience, and can be provoked in response to a cue. During a 10-min observational period in which we monitored grooming behavior, we observed a significant increase in repetitive stereotype grooming in IMC-treated mice compared to the control group (*p* = 0.004; Fig. 3*E*). In parallel, the number of grooming bouts was significantly increased in the IMC-treated group compared to the control group (*p* = 0.002; Fig. 3*F*). We next assessed food-seeking behavior using the buried cookie test (Fig. 3*G*). During this test mice are given 10 min to find a peanut butter cookie buried ∼1 cm under clean cage bedding in a novel cage following an overnight fast. Interestingly, IMC-treated mice displayed a significant increase in the time to find the hidden cookie compared to control mice, suggesting they were unable to detect the cookie odor or were less motivated to find the cookie (*p* = 1 × 10^−5^; Fig. 3*G*). To further explore whether the TMAO pathway broadly shapes olfactory perception, we conducted an extensive odor discrimination test using multiple social and non-social scents. IMC-treated mice displayed a significant decrease in time spent investigating the first two trials of tap water and first trial of almond scents compared to control mice (Water Trial 1 - *p* = 0.004; Water Trial 2 - *p* = 0.01; Almond Trial 1 - *p* = 6 × 10^−4^; Fig. 3*H*). However, IMC-treated mice did not display a deficit in investigating banana and novel mouse scents (Fig. 3, *H* and *I*). These results indicate that blocking gut microbial TMA and TMAO production with IMC can impact some, but not all, olfactory perception tests. We next investigated the treated mice’s ability to build a nest using a nestlet. Both mouse groups showed similar widths and heights of their nest during a 90-min testing period (Fig. 3, *J* and *K*). We also investigated pain tolerance using a heat stimulus. Both mouse groups displayed similar latencies to lick their hindpaw in the hot plate test (Fig. 3*L*). Next, we assessed auditory startle responses. Both groups displayed the same levels of acoustic startle response to a range of auditory stimuli from 0 to 120 dB (Fig. 3*M*). In a grip strength test, IMC-treated mice displayed a significant decrease in grip strength compared to control mice (*p* = 0.01; Fig. 3*N*). Collectively, these results demonstrate that blocking gut microbial choline TMA lyase activity with the small molecule CutC/D inhibitor IMC selectively alters innate behaviors in mice and uncovers an underappreciated link between the gut microbial TMAO pathway and olfactory perception in mice.

### Gut microbial choline TMA lyase inhibition does not alter cognitive, anxiety-like, or depression-related behaviors

**Figure 4:**
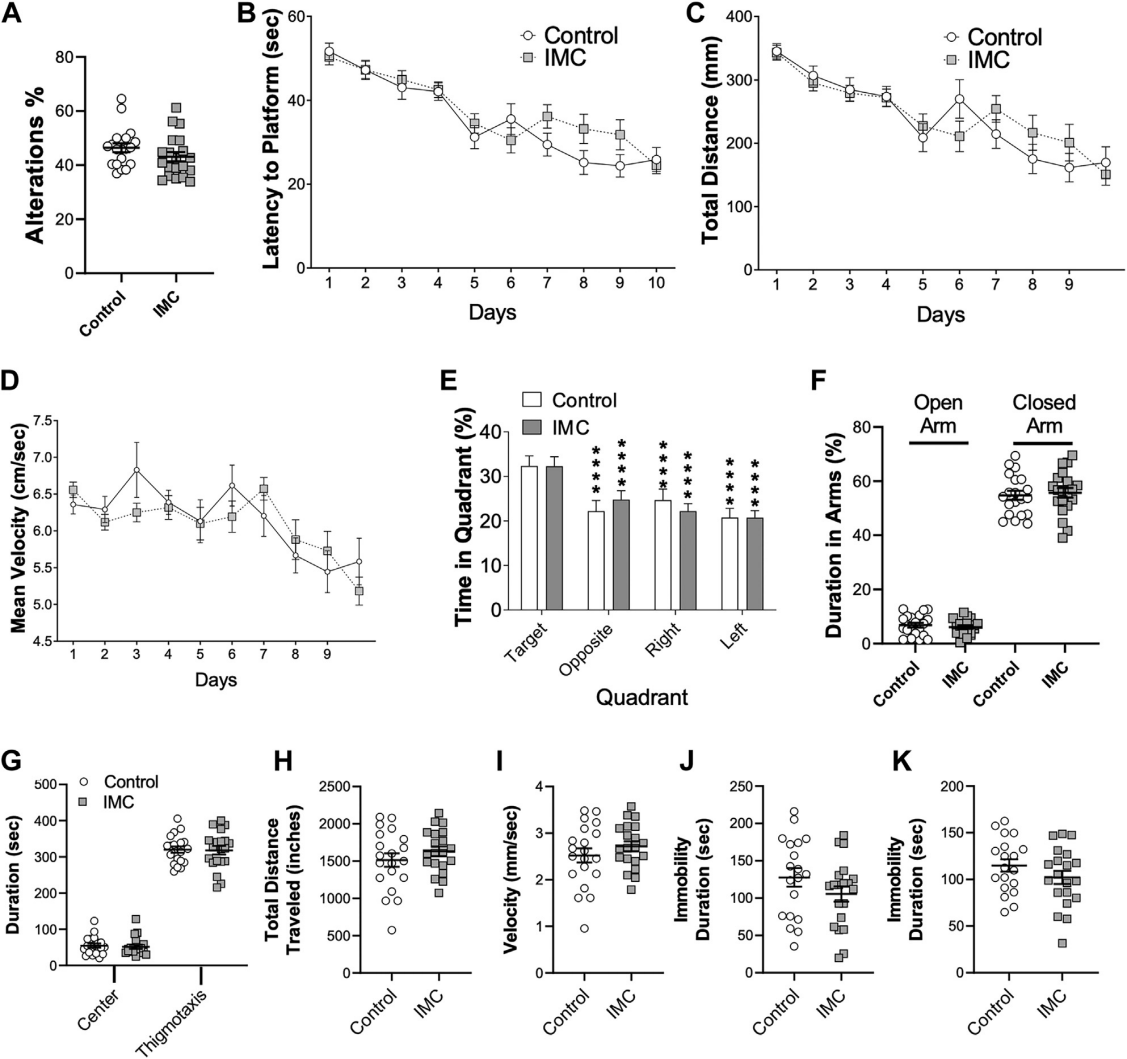
**Gut microbe-targeted choline trimethylamine lyase inhibition does not significantly alter cognitive, anxiety-like, or depression-related behaviors.** Wild-type female C57BL/6J mice were treated with or without the gut microbial CutC/D inhibitor iodomethylcholine (IMC) in the drinking water, and then subjected to a series behavioral phenotyping tests to broadly assess cognition, anxiety, and depression including (*A*) Y-maze test, (*B*–*D*) Standard Morris water maze, (*E*) Probe trial in Morris water maze, (*F*) Elevated plus maze, (*G*–*I*) Open field test, (*J*) Tail suspension test, and (*K*) Forced swim test. Data are presented as mean ± SEM; ∗*p* < 0.05; ∗∗*p* < 0.01; ∗∗∗*p* < 0.001; ∗∗∗∗*p* < 0.0001; *n* = 20 control, *n* = 20 IMC-treated for the forced swim test, all other tests had *n* = 19 control, *n* = 20 IMC-treated.

Although TMAO levels are most abundant in the olfactory bulb (Fig. 1*F*), and olfactory perception is clearly altered in mice with low TMAO levels (Fig. 3, *G* and *H*), we also wanted to more comprehensively examine other potential behavioral alterations induced by TMA lyase inhibition. Therefore, we performed a series of *in vivo* tests that report on cognition, memory, anxiety, and depression-related behaviors. To assess working memory, we used the spontaneous alteration y-maze test. IMC-treated mice displayed normal working memory shown by their ability to spontaneously explore the Y-maze arms to the same extent as control mice (Fig. 4*A*). We next assessed spatial memory using the Morris water maze method and found that IMC-treated mice showed similar latencies, distances traveled, and velocities compared to control mice (Fig. 4, *B*–*D*). Twenty-four hours after the last day of training a probe trial was conducted. Both control and IMC-treated mice displayed a preference for the target quadrant over the other quadrants (Fig. 4*E*). We next investigated anxiety in IMC-treated mice using the elevated plus maze and the open field test. In the elevated plus maze both control and IMC-treated mice showed similar durations in the open and closed arms of the maze (Fig. 4*F*). In the open field test, both control and IMC-treated mice spent similar time in the central, anxiogenic region (Fig. 4*G*). Additionally, there was no difference in distance traveled (Fig. 4*H*) or velocity (Fig. 4*I*) in the open field test. To assess depression-like behavior in the treated mice, we conducted tail suspension tests and force swim tests. In both tests, the control and IMC-treated mouse groups displayed similar durations of immobility respectively (Fig. 4, *J* and *K*).

### Social interactions are significantly altered when microbial TMA production is inhibited

**Figure 5:**
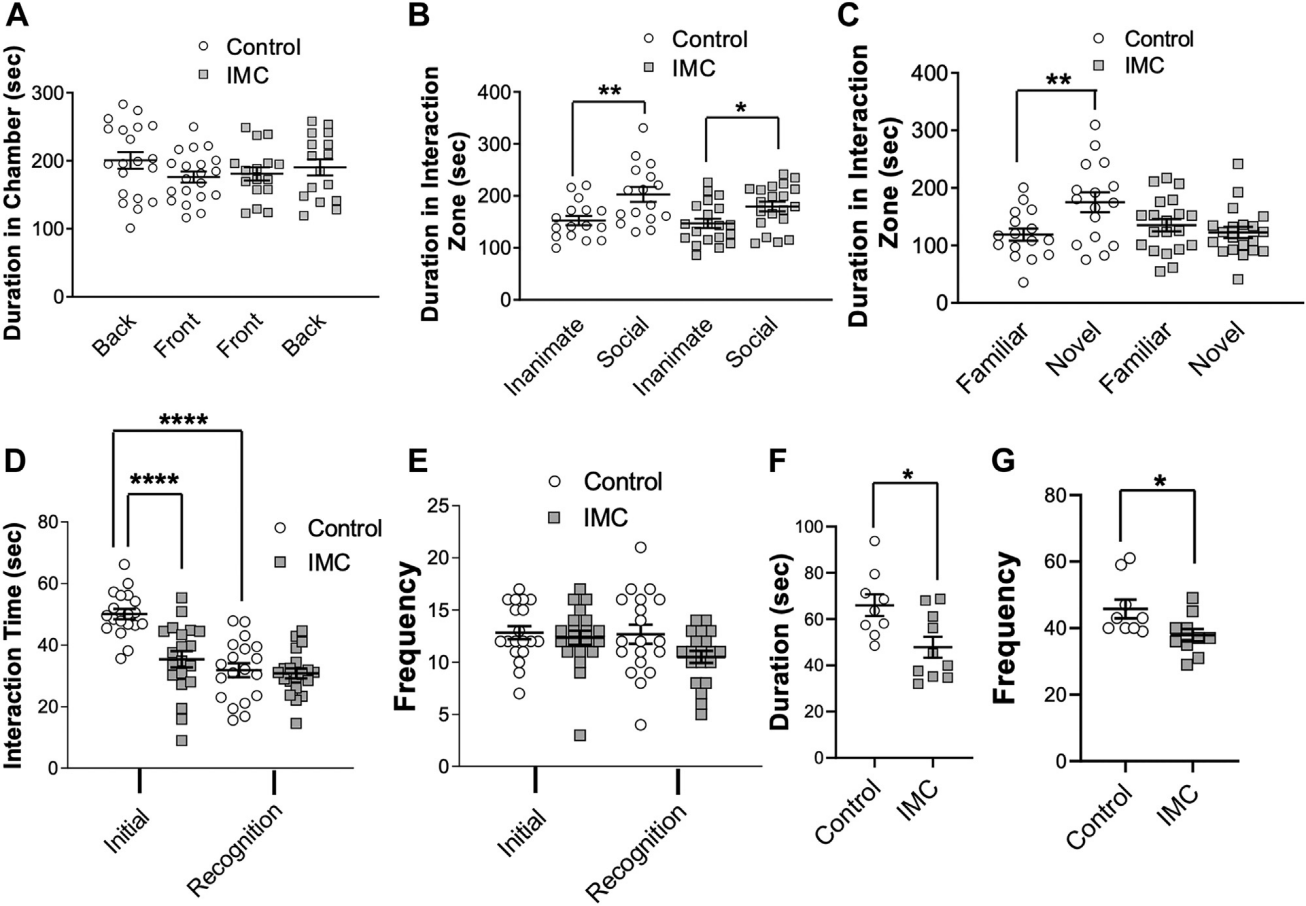
**Gut microbe-targeted choline trimethylamine lyase inhibition selectively alters some, but not all, social interaction-related behaviors.** Wild-type female C57BL/6J mice were treated with or without the gut microbial CutC/D inhibitor iodomethylcholine (IMC) in the drinking water and then subjected to a series of behavioral phenotyping tests to assess social interactions including (*A*) 3-chamber social interaction test, (*B*) Social preference test, (*C*) Social novelty test, and (*D*–*G*) Social interaction with a juvenile test. Data are presented as mean ± SEM; ∗*p* < 0.05; ∗∗*p* < 0.01; ∗∗∗∗*p* < 0.0001; *n* = 20 control, *n* = 20 treated for 3-chamber test, all other tests *n* = 19 control, *n* = 20 IMC-treated.

Social interaction is a critical behavior in animals that helps establish hierarchy and reproduction. It is important to note that social interaction is a complex behavior shaped by olfactory, visual, as well as innate cues. To understand the potential of the TMAO pathway to influence specific aspects of social interaction we performed a series of distinct behavioral tests. In the three-chamber test of sociability, IMC-treated mice displayed notable abnormalities. The initial trial of this test assessed chamber bias when neither side chamber had a stimulus target. Neither mouse group displayed a chamber bias in the initial trial (Fig. 5*A*). When one chamber contained a novel caged adult mouse and the other contained an empty cage, both the control group and IMC-treated group displayed a normal preference for social interaction (Fig. 5*B*). When one chamber contained a familiar-caged adult mouse and the other contained a novel-caged adult mouse, as expected the control group showed social novelty preference (Novel vs Familiar - Control: *p* = 0.009), yet the IMC-treated mice were unable to differentiate between the familiar and novel scenarios (Fig. 5*C*), which could support the notion of the impaired olfactory distinction between the familiar and novel mice. We next performed a social recognition memory task in which novel juvenile mice (3-week-old) were introduced to control and IMC-treated mice, and then 3 days later the same juvenile mice were reintroduced to the same experimental mouse. Control mice displayed a significant decrease in interaction time when the juvenile mouse was reintroduced (Initial vs. recognition time in control mice: *p* = 3 × 10^−5^), suggesting that the control mice recognized the juvenile as familiar (Fig. 5*D*). IMC-treated mice also displayed clear interaction deficits (control vs IMC-treated: *p=*7 × 10^−7^) during the initial trial when compared to the controls (Fig. 5*D*). However, both groups showed a similar number of social interaction bouts (Fig. 5*E*). Finally, we assessed reciprocal social interaction by pairing mice in either the control or the treated group with another mouse in the same group for an initial encounter. IMC-treated mouse pairs displayed decreased social interaction (Fig. 5*F*; Control vs Treated: *p* = 0.01) and a number of social interaction bouts (Fig. 5*G*; Control vs Treated: *p* = 0.03) compared to the control group. Collectively, these results show that reducing bacterial TMA production using the choline TMA lyase inhibitor IMC alters some, but not all, social interactions in mice.

### Mice treated with IMC display decreased TMA and TMAO systemically but only decreased TMAO in the olfactory bulb

**Figure 6:**
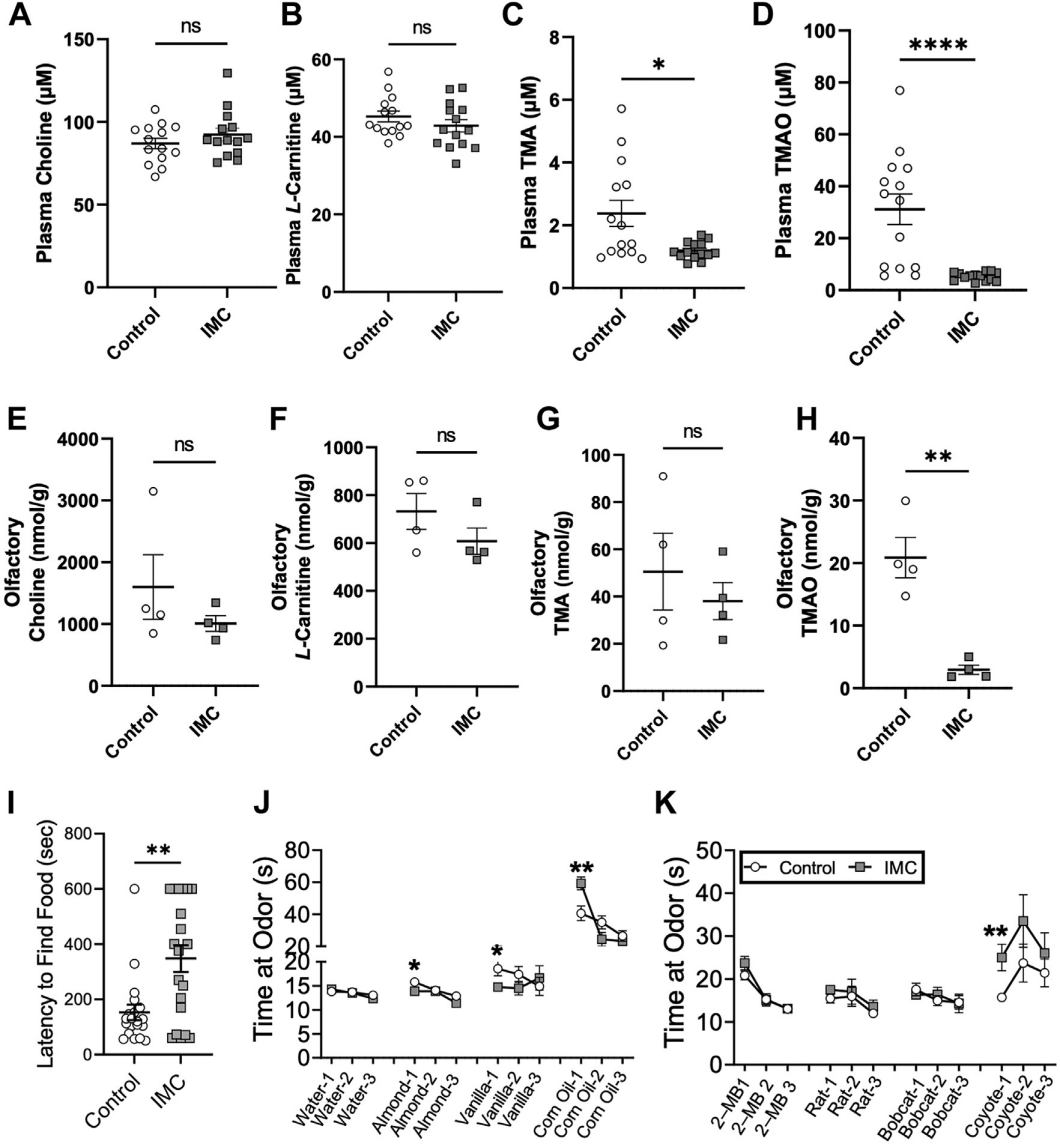
**TMA lyase inhibition alters olfactory TMAO, but not TMA and consistently shows defects in olfactory behavior**. Female wild type were treated with or without the gut microbial CutC/D inhibitor iodomethylcholine (IMC) in the drinking water, and then subjected to plasma and tissue metabolomics as well as the olfactory cookie test and a broadened olfactory discrimination test compared to that in Figure 2. *A*–*D*, Plasma and (*E*–*H*) olfactory levels of trimethylamine N-oxide (TMAO) pathway co-metabolites (*A* and *E*) choline, (*B* and *F*) L-carnitine, (*C* and *G*) trimethylamine (TMA), and (*D* and *H*) TMAO were quantified *via* LC-MS/MS. Latency to find the cookie was significantly increased in FMO3^TG^ mice (*I*). Attractant (*J*) and deterrent (*K*) odor discrimination testing was performed. Data are presented as mean ± SEM and represent *n* = 20 for the plasma metabolites and behavioral testing (panels *A*–*D*, *I*–*K*), *n* = *4* for tissue metabolomics (*E*–*H*).

To better understand the effects of choline TMA lyase inhibition on the distribution of TMAO and its co-metabolites in the olfactory bulb and a broader range of olfactory discrimination testing, we conducted additional studies with animals treated with or without IMC (Fig. 6). In the olfactory bulb, we confirmed that TMAO is significantly reduced in IMC-treated mice *versus* control animals (*p* = 0.0016; Fig. 6*H*). In contrast, choline, *L-*carnitine, and TMA are unchanged (Fig. 6, *E* and *F*) in the olfactory bulb. In the buried cookie test, this replication cohort (*p* = 0.0012; Fig. 6*I*) mirrored the findings of previous studies (Fig. 3*G*). Here, we next expanded the range of scents used for olfactory discrimination testing to include both attractant and deterrent scents. In this cohort, IMC-treated mice showed no difference in any of the trials with distilled water (Fig. 6*J*) as opposed to tap water, which was originally tested (Fig. 3*H*). Also, there was a similar decrease in the time at almond odor in the IMC-treated mice (Almond Trial 1 - *p* = 0.0417; Fig. 6*J*) as in prior studies (Fig. 3*H*). In addition, there were significant decreases in time at vanilla and corn oil odors in the IMC-treated mice (Vanilla Trial 1 - *p* = 0.0242, Corn Oil Trial 1 - *p* = 0.0039; Fig. 6*J*). In testing deterrent scents, there was an increased time at odor in IMC-treated mice exposed to coyote urine but not 2-methylbutyrate (2 MB), rat urine, or bobcat urine (Coyote Trial 1 - *p* = 0.0067; Fig. 6*K*). Collectively, these data confirm that gut microbial production of TMA is associated with altered olfactory perception to diverse odor stimuli.

## Discussion

There are numerous examples of gut bacteria-derived metabolites that can cross the BBB, and several examples of where specific microbial metabolites can impact behavioral phenotypes and disease pathogenesis in the central nervous system (25, 26, 27, 28, 29, 30, 31). Bacteria can also produce many neuroactive compounds that can influence systemic inflammation, neuroinflammation, and in some cases signal directly to parenchymal cells in the brain. In fact, bacteria residing within the gastrointestinal tract can be considered as potentially the largest endocrine organ in our body, and our understanding of gut-brain axis endocrinology is only in its infancy. Among the many thousands of bacterial metabolites, several have emerged as particularly compelling in modulating brain health including short-chain fatty acids (SCFA), tryptophan metabolites, and indoles (25, 26, 27, 28, 29, 30, 31). However, one gut microbe-derived metabolite that has been poorly studied in the field of neuroscience is TMAO. There is now emerging evidence that circulating levels of TMAO are associated with several disruptions of typical brain function including Alzheimer’s disease (40, 41, 42, 43), Parkinson’s disease (44, 45), and ischemic stroke (46, 47, 48, 49, 50, 51). It is interesting to note that all of these disorders of the brain are closely associated with anosmia or altered olfactory perception (62, 63, 64, 65). This study demonstrates for the first time that TMAO is particularly enriched in the murine olfactory bulb, and inhibition of TMAO production at the level of the gut microbiome (*i.e.*, IMC treatment) impacts the olfactory perception of food and social cues.

It has long been assumed that TMAO is primarily made in the liver, where *Fmo3* is abundantly expressed (58). However, the levels of TMAO throughout the brain are very similar to those found in the liver, with the exception being the olfactory bulb where TMAO is 2-fold higher than what is seen in the liver (Fig. 1*F*). The fact that the level of TMAO is highest in the olfactory bulb compared to other brain regions or the liver potentially indicates that the olfactory bulb may have unique TMAO transporters, synthesizing enzymes like FMO1 and FMO3, or low turnover of TMAO. Additional work will be needed to understand how TMAO can accumulate locally in the olfactory bulb, and whether this can be manipulated to impact olfactory perceptive issues that arise due to neurodegenerative diseases (40, 41, 42, 43, 44, 45), stroke (46, 47, 48, 49, 50, 51), and commonly with infectious diseases such as COVID-19 (63). Given the key role that the TMAO pathway plays in cardiovascular and related metabolic diseases (51, 52, 53, 54, 55, 56, 57, 58), there are continuing efforts to develop pre-biotic, probiotic, and even bacterially targeted small molecule drugs (like IMC used here) to lower circulating TMAO levels. It is an intriguing possibility that these same TMAO-lowering therapeutic approaches may also be leveraged to restore olfaction in diverse anosmia etiologies in humans. Based on these findings, it will be important to track olfactory effects as these pharmacological agents are advanced into human trials.

Although gut microbes produce a vast array of small molecule metabolites, a commonly overlooked characteristic of many bacterially generated metabolites is their strong odor (64, 65, 66). In fact, many of the odors we commonly associated with food spoilage, human body odor, and death actually originate from small molecule metabolites made by bacteria (64, 65, 66). Bacterially-derived small molecules including biogenic amines (TMA, cadaverine, putrescine, spermidine, etc.), sulfur compounds (hydrogen sulfide, dimethyl sulfide, methyl thioacetate, etc.), alcohols (heptanol, butanol, 3-methyl-1-butanol, etc.), ethyl esters (ethylacetate, ethylbutanoate, ethyloctanoate, etc.), ketones (acetoin, diacetyl, 3-octanone, etc.), and SCFAs (acetate, butyrate, hexanoate, etc.) have unique odors that are detected by specific host olfactory receptor systems (64, 65, 66, 67, 68, 69). It is interesting to note that bacterially derived TMA smells like rotting fish, and humans with mutations in the TMA to TMAO converting enzyme FMO3 have a disease known as “fish odor syndrome” or TMA (63). However, TMAO does not have a similar fishy odor and does not activate the same olfactory receptor as TMA (67, 68, 69). The only known TMA-sensing host receptor is the G protein-coupled receptor trace amine-associated receptor 5 (TAAR5) (67, 68, 69). Notably, Taar5 is expressed in the olfactory epithelium in C57BL/6 mice where it can regulate TMA-driven mating behaviors (67). Because TAAR5 is central to sensing TMA in the olfactory epithelium for murine mating behaviors (67), it may be involved in diverse olfactory responses in this study. Additional work is needed to understand how TMA and or TMAO is sensed in the olfactory system, and how this sensing may secondarily impact other olfactory circuits for scents such as almond, vanilla, and corn oil.

Small molecule chemical communication is widely used in interspecies crosstalk for the dominant host to obtain essential life-or-death information from the surrounding environment. Interestingly, small molecule metabolites originating from bacteria can have profound effects on feeding preference *versus* food avoidance in the host. For instance, foul-smelling metabolites such as cadaverine, putrescine, and TMA, which are bacterial byproducts of rotting flesh, typically elicit anorexia and food avoidance (64, 65, 66); whereas, more pleasant-smelling bacterial metabolites such as ethyl acetate or phenylethyl alcohol play a major role in driving our appetite towards foods where they are abundant such as citrus fruits (64, 65, 66). These types of microbe–host olfactory circuits are mediated predominantly *via* a subclass of chemosensory G protein-coupled receptors known as olfactory or trace amine-associated receptors. As gut-brain axis research advances, it will not only be important to identify the host receptor systems that sense bacterial metabolites but also to design studies that take into account the multi-faceted interactions between dietary inputs (*i.e.* substrates available to gut microbes), bacterial metabolic capacity (*i.e.* are the correct bacterial strains present in the gut to produce key metabolites), and brain regional-specific uptake and metabolism of bacterial products. Our findings support the exciting possibility that rationally designed “psychobiotic” therapies that include diet, microbe, and host constraints can be used to improve anosmia and the diverse array of diseases that arise in the central nervous system.

## Experimental procedures

### Animals

Unless otherwise specified, age-matched female C57BL/6J mice were obtained from Jackson Laboratories. Inhibition of the gut microbial choline TMA lyase (*CutC/D*) was achieved by synthesizing and providing an inhibitory dose (0.06% in the drinking water) of iodomethylcholine (IMC) as we have previously described (58). Following all behavioral assays in control *versus* IMC study, plasma was collected by cardiac puncture. Thereafter, mice were slowly perfused with 10 ml of saline, and tissues and brain regions were collected, flash-frozen, and stored at −80 °C until the time of analysis. All mice were maintained in an Association for the Assessment and Accreditation of Laboratory Animal Care, an International-approved animal facility, and all experimental protocols were approved by the Institutional Animal Care and use Committee of the Cleveland Clinic.

### Measurement of plasma and tissue TMAO and co-metabolites

Stable isotope dilution high-performance liquid chromatography with online tandem mass spectrometry (LC–MS/MS) was used for quantification of levels of TMAO, TMA, choline, carnitine, and *γ*-butyrobetaine in plasma, as previously described (70). Their d_9_(methyl)-isotopologues were used as internal standards. Tissue (1 mg tissue is equivalent to 1 μl in volume) was homogenized with 20 volumes of methanol containing d_9_(methyl)-isotopologue internal standard mix. LC–MS/MS analyses were performed on a Shimadzu 8050 triple quadrupole mass spectrometer. TMAO and its d_9_(methyl)-isotopologue, along with other metabolites, were monitored using multiple reaction monitoring of precursor and characteristic product ions as follows: m/z 76.0 → 58.1 for TMAO; m/z 85.0 → 66.2 for d_9_-TMAO; m/z 60.2 → 44.2 for TMA; m/z 69.0 → 49.1 for d_9_-TMA; m/z 104.0 → 60.1 for choline; m/z 113.1 → 69.2 for d_9_-choline; m/z 118.0 → 58.1 for betaine; m/z 127.0 → 66.2 for d_9_-betaine. Series concentrations of the standard mix were mixed with the internal standard mix to prepare calibration curves.

### Behavioral phenotyping overview

Behavioral tests in mice treated with gut microbe-targeted choline TMA lyase inhibitor IMC were performed on a cohort of 40 age-matched female (n = 20 control untreated and n = 20 IMC-treated), mice. Mice either received normal water or water-containing IMC starting at 6 weeks of age and continuing throughout the comprehensive behavioral testing. All behavior was performed during the light cycle of the mouse. Behavioral testing started at ∼ 2 months of age in the order of least to most stressful. The order was as follows: elevated plus maze, open field, grooming, 3-chamber social interaction test, spontaneous y-maze, grip strength, social interaction with juvenile, olfactory cookie test, olfactory discrimination test, nesting, Morris water maze, startle, tail suspension, force swim test, social interaction with match pairs, and hotplate sensitivity. Behavioral results are not described in the order they were tested in an effort to ease the presentation and interpretation of the data.

### Morris water maze

The Morris water maze task was conducted essentially as described previously (71, 72) A 48” diameter, white, plastic, circular, heated pool was filled to the depth of 23.5” with 22 °C ± 1 °C water made opaque with gothic white, nontoxic, liquid tempera paint in a room with prominent extra-maze cues. Mice were placed in one of the four starting locations facing the pool wall and allowed to swim until finding a 15 cm diameter clear platform for 20 s before being removed to the home cage. If mice did not find the platform within 60 s, they were guided to the platform by the experimenter and remained on the platform for 20 s before being removed to the home cage. Latency to reach the platform, distance traveled to reach the platform, swim speed, and time spent in each of four quadrants were obtained using automated video tracking software from Noldus (Ethovision XT13, Leesburg VA). Mice were trained with 4 trials/day with an intertrial interval of 1 to 1.5 min for 10 consecutive days between 10 AM and 3pm. A probe trial (free swim with the submerged platform removed) was performed as the first trial of the day on days 11. Percent time spent in the target quadrant was calculated. Latency to platform, distance to platform, swim speed, and time spent in the target quadrant was analyzed.

### Elevated plus maze

The elevated plus maze task was conducted essentially as described previously (73) Mice were placed in the center of a black, plexiglass elevated plus maze (each arm 33 cm long and 5 cm wide with 25 cm high walls on closed arms) in a dimly lit room for 5 min. Automated video tracking software from Noldus Ethovision XT13 (Leesburg VA) was used to track time spent in the open and closed arms, number of open and closed arm entries, and number of explorations of the open arm (defined as placing head and two limbs into open arm without full entry).

### Open field

The open field was conducted as described previously (71, 72). Briefly, mice were placed along the edge of an open arena (44 × 44 × 44 cm) and allowed to freely explore for 10 min. Time spent in the center of the arena 15 × 15 cm as well as locomotor activity were measured. Mice were monitored using Noldus EthoVision XT14 (Leesburg VA).

### Grooming

Mice were placed in a clean novel home cage without the bedding, and time spent grooming the face, head, body, or tail was measured for 10 min. The number of grooming bouts that lasted >1 s was also recorded.

### Hotplate sensitivity

This test was performed as described previously (74). Mice were placed on a black, anodized plate that was held at a constant temperature of 52 °C (Ugo Basile hot/cold plate) covered with a Plexiglas enclosure. Thereafter, mice were removed after the first hindpaw lick or after 30 s if no response was elicited.

### Social behavior

Social interaction with a juvenile was performed in a novel empty home cage under dim white light as described previously (71, 72). Briefly, following a 15-min habituation, a test mouse was placed in a novel empty cage with a juvenile stimulus mouse (BALB/cJ juvenile mouse; 3 weeks of age; Stock #000651 Jackson Labs) and allowed to directly interact for 2 min. Interaction was scored by observing the duration and number of times the test mouse-initiated contact with or sniffed the juvenile mouse. Contact was considered as any part of the body touching the other mouse. Three days later the same test mouse and juvenile mouse were paired again in a novel clean cage for 2 min and scored in the same manner. Social interaction with match pairs was performed by pairing a treated mouse with another treated mouse and an untreated mouse with another untreated mouse. Matched pairs were derived from separate cages and were never previously housed together. Mouse pairs were placed at separate ends in an open field arena (44 × 44 × 44 cm) and allowed to interact for 5 min 3-chamber social interaction test was performed using a three-chambered box as described previously (71, 72) and based to a large extent on the original descriptions (75, 76). This test consisted of three 10-min trials. During the first trial, the mouse was allowed to explore the 3-chamber box in which each end-chamber contained an empty cage (upside-down pencil holder). In the second trial, the 3-chamber box contained a novel stimulus mouse under a cage in one of the end-chambers and an inanimate object (ping pong ball) under a cage in the opposite end-chamber. The test mouse was free to choose between a caged inanimate object and a caged, social target. For the third trial, the test mouse was free to choose between a caged novel social target (novel mouse) *versus* the same caged mouse in trial 2 (familiar social target). Locations of inanimate targets and social targets were counterbalanced, and mice were placed back into the home cage for very brief intervals between trials. Total duration in chamber and interaction zone was recorded using video-tracking and automated Noldus EthoVision XT 14 (Leesburg VA).

### Spontaneous alteration in Y-Maze

Spontaneous alteration in Y-Maze was performed as previously described (77). Briefly, a mouse was placed into one arm of the Y-maze facing the center (14”). Video tracking was used to record the spontaneous behavior of each mouse for 10 min. Zone (*i.e.* arm) alteration was later analyzed using Noldus EthoVision XT14, to determine when a mouse entered 3 consecutive different arms of the maze. Spontaneous alteration % is calculated as follows: Alteration % = #spontaneous alterations/total number of arm entries −2 x 100.

### Startle test

We used a variation of the protocol of Dulawa and Geyer (78, 79). Briefly, mice were placed inside a cylinder mounted atop a piezoelectric accelerometer that detected and transduced mouse movements inside the startle chambers (San Diego Instruments, San Diego, CA, USA). Acoustic stimuli varying in decibels (dB) was delivered by high-frequency speakers mounted 33 cm above the cylinder. To test startle response mice were exposed to stimuli ranging from 0 to 120 dB (0, 80, 90, 100, 110, and 120 dB) and the amplitude of the startle responses was obtained in mV.

### Force swim test

The force swim test was performed as described previously (80). Briefly, mice were placed into 4L glass beakers filled with 3L of water between 22 to 25 °C for 6 min. Video-tracking software, Noldus EthoVision XT15 was used to record each mouse. The last 4 min of the trial were analyzed for mobility. Mobility is defined as any movements other than those necessary to balance the body and keep the head above the water.

### Tail suspension test

The tail suspension test was performed as previously described (81). Briefly, tape 17 cm long was used to suspend mice by their tails for 6 min while being video recorded for analysis. During analysis the amount of time a mouse spent immobile in the last 4 min was recorded.

### Nesting

Nesting behavior was performed in a well-lit room by placing a mouse in a novel home cage with a cotton nestlet (5.5 × 5.5 × 0.5 cm) but no bedding. Height and width of the nests were measured at 30, 60, and 90 min.

### Olfactory discrimination test

This test was performed as described previously (82). Briefly, a set of non-social attractant odors (almond, banana, vanilla, corn oil, and water), non-social deterrent odors (2-methylbutyrate, rat urine, bobcat urine, and coyote urine), and two social odors were prepared. Three 6” cotton-tipped applicators containing a single scent were prepared for each of the five odors. Each odor was presented 3 times in a row for 2 min. The cotton applicator was tapped to the wire cage lid on an empty clean home cage so that the cotton tip was hanging just below the wire lid. The odors were presented in the following order: water, almond, banana, social odor 1, and social odor 2. All trials were video recorded and the amount of time each mouse spent exploring the cotton tip was recorded.

### Olfactory cookie test

One day prior to testing, food was removed from each mouse’s home cage. The following day, a small portion of peanut butter cookie (Nutter Butter, Nabisco) was placed randomly ∼1 cm under the bedding in a novel clean mouse home cage (72, 83). A test mouse was taken from it’s home cage and placed into the cage with the hidden cookie an allowed 10 min to find the cookie. Latency to find the cookie was measured and recorded.

### Analysis of gene expression using quantitative real-time polymerase chain reaction (qRT-PCR)

qRT-PCR analysis was conducted as described previously (1). cDNA was prepared using qScript SuperMix (95,048, QuantaBio). SYBR Fast reagent (4,385,618, Applied Biosystems) on an Applied Biosystems StepOne Plus machine was used. Data were analyzed using the ΔΔCT method, and expression was normalized such that the average expression of *Fmo1* in the brainstem (Fig. 2). All primer sequences are listed here:

*Cyclophilin A* Fwd.

5′-GCGGCAGGTCCATCTACG-3’

*Cyclophilin A* Rev.

5′-GCCATCCAGCCATTCAGTC-3’

m*Fmo1* Fwd.

5′-GATGACCTCCTGACCTCTATCA-3’

m*Fmo1* Rev.

5′-GGTACATGGGCCAAAGAAGA-3’

m*Fmo2* Fwd.

5′-CTGCTTCGAAAGGACTGAAGA-3’

m*Fmo2* Rev.

5′-GGTAATGACAGAGCGGTAGATG-3’

m*Fmo3* Fwd.

5′-CCCACATGCTTTGAGAGGAG-3’

m*Fmo3* Rev.

5′-GGAAGAGTTGGTGAAGACCG-3’

m*Fmo4* Fwd.

5′-TCACAGAGACAGAAGGCAAAC-3’

m*Fmo4* Rev.

5′-GGAAAGGACTCCAGAGGTAAATG-3’

m*Fmo5* Fwd.

5′-CCTTCAGAACCTGATGAGACTG-3’

m*Fmo5* Rev.

5′-AGAGAGAGAGAGAGAGAGAGAGA-3’

### Statistical analyses

Measurements are means ± SEM of biological replicates. All data were analyzed in GraphPad Prism software (version 9.1.2; GraphPad Software, Inc). Group differences were analyzed using student’s *t* test Differences were considered significant at *p* < 0.05.

## Data availability

All data are contained within this manuscript.

## Conflict of interest

W. J. M., K. E. K., T. C. J., A. H., S. C., L. J. O., R. B., D. J. S., A. B., A. L. B., O. R., J. D. L., and J. M. B. all declare no competing financial interests. Z. W. and S. L. H. report being named as co-inventor on pending and issued patents held by the Cleveland Clinic relating to cardiovascular diagnostics and therapeutics. S. L. H. also reports being a paid consultant for Zehna Therapeutics. S. L. H reports having received research funds from Procter & Gamble, Zehna Therapeutics and Roche Diagnostics. Z. W. and S. L. H. report being eligible to receive royalty payments for inventions or discoveries related to cardiovascular diagnostics or therapeutics from Cleveland Heart Lab, and Procter & Gamble, and S. L. H. from Zehna Therapeutics.
